# Cradle-to-Gate Life-Cycle Assessment of a Waste Printed Circuit Board-Derived Adsorbent: Production Impacts Normalized by Heavy-Metal Adsorption Capacity

**DOI:** 10.3390/molecules31183141

**Published:** 2026-09-08

**Authors:** Junaid Saleem, Zubair Khalid Baig Moghal, Gordon McKay

**Affiliations:** Division of Sustainable Development, College of Science and Engineering, Hamad Bin Khalifa University, Qatar Foundation, Doha P.O. Box 5825, Qatar

**Keywords:** ion adsorbent, PCB, e-waste, LCA, adsorption, printed circuit board

## Abstract

A cradle-to-gate life-cycle assessment (LCA) was conducted for an activated adsorbent produced from the non-metallic fraction (NMF) of waste printed circuit boards. The primary production reference was 1 kg of dry activated non-metallic fraction (ANMF) produced. Cradle-to-gate production impacts were subsequently normalized using previously reported equilibrium adsorption capacities for Pb^2+^, Cu^2+^, and Zn^2+^ to obtain performance-normalized production impacts per kilogram of theoretical metal uptake. Environmental impacts were quantified using the ReCiPe 2016 Midpoint (H) method together with cumulative energy demand (CED). Production of 1 kg of ANMF resulted in a climate change (CC) impact of 5.77 kg CO_2_-eq and a CED of 140.03 MJ. After adsorption-capacity normalization, the CC impacts were 8.44, 31.35, and 43.05 kg CO_2_-eq kg^−1^ metal uptake, while the corresponding CED values were approximately 205, 761, and 1045 MJ kg^−1^ metal uptake for Pb^2+^, Cu^2+^, and Zn^2+^, respectively. Commercial activated carbon (AC) exhibited slightly lower production impacts, but its lower representative Pb^2+^ adsorption capacity resulted in substantially higher normalized production burdens. These normalized indicators represent cradle-to-gate production impacts scaled by adsorption performance and do not include wastewater-treatment use-stage operations, regeneration, or management of spent adsorbent. The results demonstrate that adsorption performance substantially influences the interpretation of production-related environmental burdens and should therefore complement conventional mass-based assessment of adsorbents.

## 1. Introduction

Heavy-metal contamination of water remains a major environmental challenge because of the persistence, toxicity, and bioaccumulation of metals such as Pb^2+^, Cu^2+^, and Zn^2+^ [1,2]. Adsorption is widely recognized as one of the most effective treatment technologies owing to its operational simplicity, high removal efficiency, and ease of regeneration [3,4,5]. Consequently, considerable research has focused on developing low-cost adsorbents from waste resources to replace conventional commercial activated carbon (AC) while simultaneously promoting waste valorization and circular resource utilization. Among these, waste printed circuit board (WPCB) non-metallic fractions (NMFs), which are generated in large quantities during electronic waste recycling [6,7,8,9], have received increasing attention as a valuable secondary resource for sustainable material recovery and waste valorization [10,11].

Beyond adsorption performance, the environmental sustainability of adsorbent production has become an increasingly important consideration. Life-cycle assessment (LCA) has therefore been widely applied to quantify the environmental impacts associated with adsorbent production, enabling different materials and preparation routes to be compared on a consistent basis [12,13]. Previous studies have primarily focused on reducing production-related environmental burdens through process optimization, selection of activation chemicals, and improvements in manufacturing efficiency [14,15,16]. These assessments are almost universally reported per kilogram of adsorbent produced, thereby evaluating the environmental burdens associated with manufacturing rather than pollutant removal.

However, the practical objective of an adsorbent is not to produce one kilogram of material but to remove a given quantity of pollutant. Adsorption capacity directly determines the amount of adsorbent required to achieve this objective and therefore influences the associated environmental burdens during application. As a result, two adsorbents with similar—or even different—production impacts may exhibit substantially different environmental performances when evaluated according to the amount of pollutant uptake. Despite this fundamental relationship, adsorption performance is rarely incorporated into the functional unit of LCA [17,18], and environmental comparisons remain predominantly based on the mass of adsorbent produced. Consequently, conventional mass-based assessments may not fully represent the environmental sustainability of adsorbents intended for wastewater treatment.

Beyond adsorbent production, LCA studies related to WPCBs have primarily focused on PCB manufacturing, supply-chain decarbonization, waste-management strategies, and the recovery of valuable metals [19,20,21,22]. These studies have identified the major environmental hotspots associated with raw-material production, energy-intensive manufacturing, recycling processes, and end-of-life management, thereby supporting the development of more sustainable PCB value chains. Comparatively less attention has been given to the non-metallic fraction (NMF), despite it representing a substantial by-product of WPCB recycling. For example, Ozkan et al. [23] conducted an LCA of PCB manufacturing and identified etching operations and copper-containing wastewater sludge disposal as major environmental hotspots, highlighting opportunities for improving manufacturing sustainability.

Activated non-metallic fraction (ANMF) adsorbents derived from WPCB NMFs have recently demonstrated excellent heavy-metal adsorption performance, offering a promising route for simultaneously valorizing electronic waste and treating contaminated wastewater [24,25,26,27,28,29]. However, to the best of our knowledge, no LCA has been reported for WPCB NMF-derived adsorbents.

A limited but growing number of LCA studies have incorporated adsorption performance into the assessment of adsorbents using both material-based and pollutant-removal-based reference metrics. Previous studies have evaluated activated-carbon production using mass- and performance-based metrics, employed pollutant-removal-based functional units for wastewater-treatment materials, and extended such assessments to regeneration and end-of-life scenarios [17,18,30]. Accordingly, adsorption-capacity normalization itself is not introduced here as a new LCA methodology.

The application-specific contribution of the present study is the cradle-to-gate environmental assessment of WPCB-NMF-derived ANMF and the evaluation of how its reported equilibrium adsorption capacities for Pb^2+^, Cu^2+^, and Zn^2+^ influence the interpretation of production-related environmental burdens. The production impacts are additionally benchmarked against commercial activated carbon to illustrate the importance of considering adsorption performance together with conventional production-based indicators.

## 2. Results and Discussion

The environmental results presented in this section are based on a cradle-to-gate assessment of ANMF production, with 1 kg of dry ANMF as the primary production reference. The resulting production impacts were subsequently normalized using the reported equilibrium adsorption capacities for Pb^2+^, Cu^2+^, and Zn^2+^ to examine the influence of adsorption performance on the environmental burden per unit of theoretical metal uptake. The detailed system boundary, life-cycle inventory, background datasets, normalization procedure, and sensitivity assumptions are described in Section 3.

### 2.1. Cradle-to-Gate Production Impacts of ANMF

**Figure 1:**
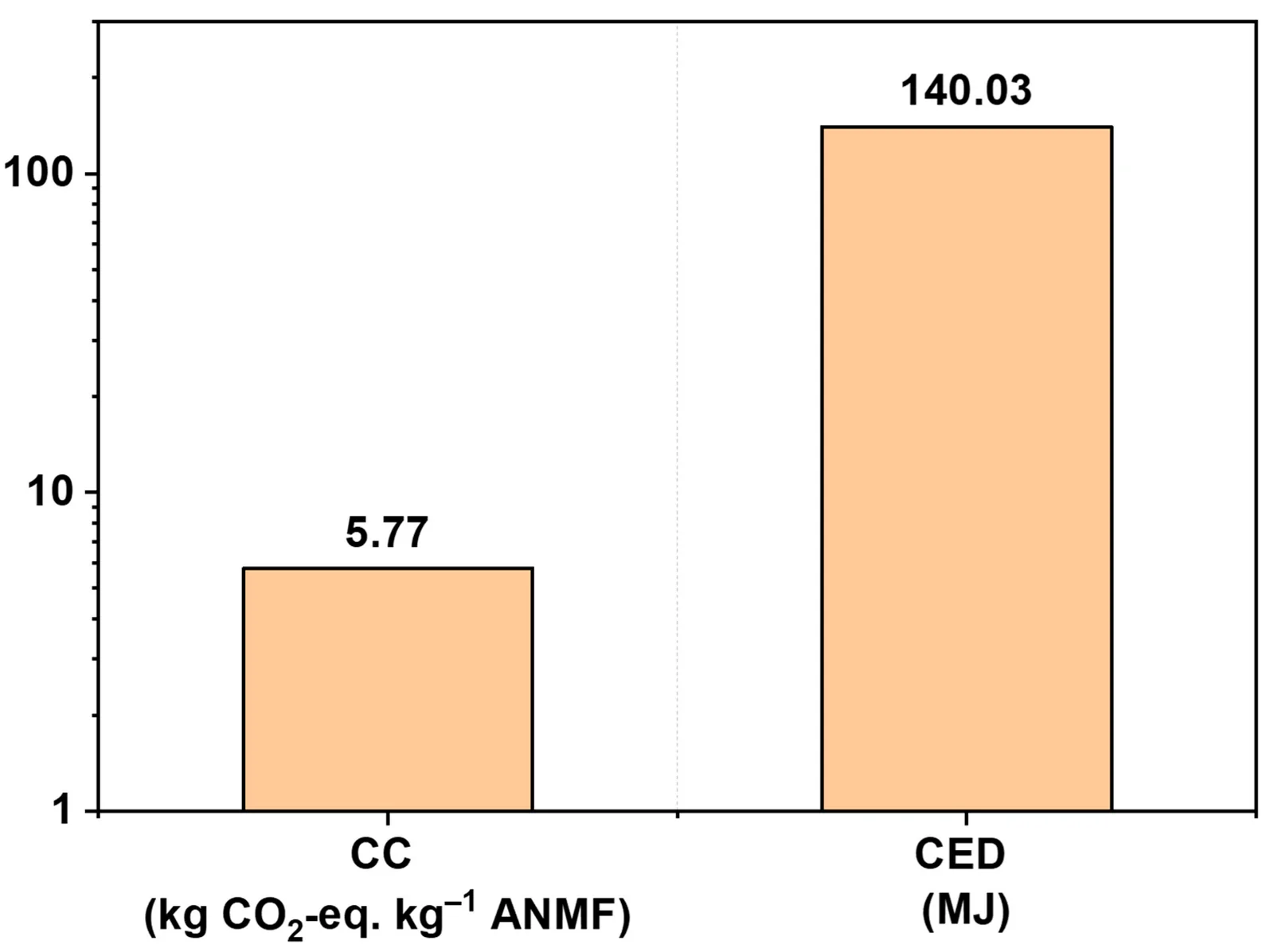
Climate change (CC) impact and cumulative energy demand (CED) associated with the production of ANMF.

Figure 1 presents the cradle-to-gate production impacts of ANMF before adsorption-capacity normalization. The production of 1 kg of ANMF resulted in a climate change (CC) impact of 5.77 kg CO_2_-eq and a cumulative energy demand (CED) of 140.03 MJ. These values represent the environmental impacts associated with adsorbent manufacture and provide the baseline inventory from which all normalized impacts were subsequently calculated. Activation conditions, chemical inputs, and energy use are also commonly reported as key contributors to the environmental burdens of adsorbent production [31].

Although these production-based indicators are useful for describing the environmental impacts of ANMF manufacture, they do not reflect the environmental efficiency of the adsorbent during pollutant removal. In adsorption applications, the relevant treatment objective is not the production of 1 kg of adsorbent, but the removal of a defined quantity of contaminant. Therefore, an adsorbent with a slightly higher production impact may still exhibit lower environmental burdens during application if its adsorption capacity is sufficiently high. This distinction is particularly important for ANMF because its heavy-metal uptake capacity differs substantially among Pb^2+^, Cu^2+^, and Zn^2+^. Consequently, the production-based impacts shown in Figure 1 were used as the starting point for functional normalization, allowing the environmental performance of ANMF to be evaluated per kilogram of heavy metal uptake.

The contribution analysis showed that process electricity and KOH production dominated the cradle-to-gate impacts of ANMF production. Electricity accounted for 52.8% of the total CC impact and 47.6% of CED, while KOH production contributed 46.3% and 51.4%, respectively. Among the electricity-consuming operations, thermal dehydration was the largest contributor, accounting for 33.0% of total CC and 29.7% of total CED, followed by thermochemical conversion at 12.5% and 11.3%, respectively. Transport, water supply, nitrogen, and wastewater treatment collectively contributed less than 1% of either indicator.

### 2.2. Performance-Normalized Production Impacts of ANMF

The environmental performance of adsorbents is conventionally reported per kilogram of material produced. However, in practical wastewater treatment, the quantity of adsorbent required is governed by adsorption capacity rather than production alone. Consequently, expressing environmental impacts on the basis of pollutant removal provides a more application-relevant assessment of adsorbent sustainability.

**Figure 2:**
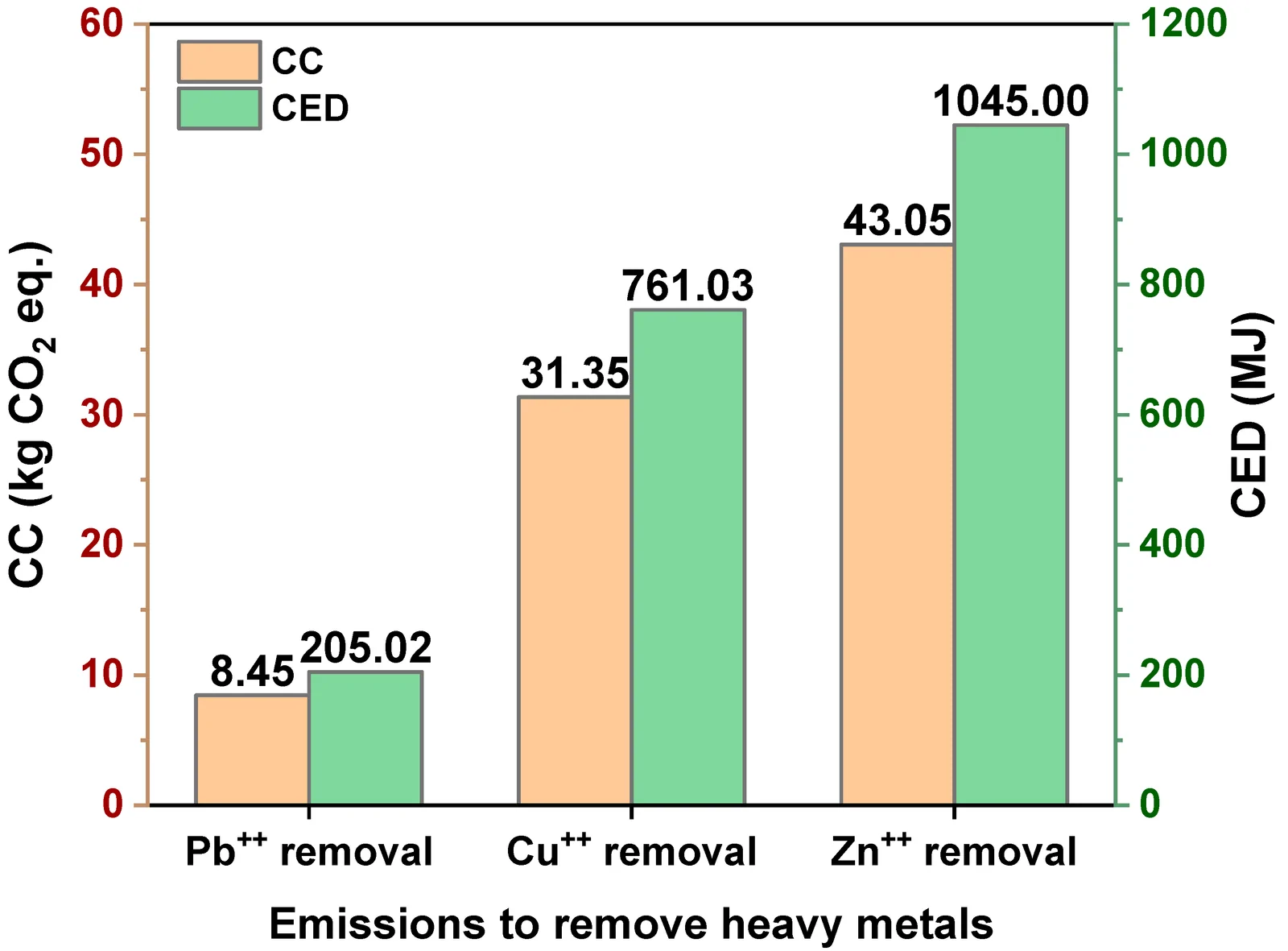
Adsorption-capacity-normalized production impacts for Pb^2+^, Cu^2+^, and Zn^2+^: Adsorption-capacity-normalized production impacts for Pb^2+^, Cu^2+^, and Zn^2+^ showing CC and CED.

Using the adsorption-capacity normalization described in Section 2.4, the cradle-to-gate production impacts were normalized using the corresponding equilibrium capacities for Pb^2+^, Cu^2+^, and Zn^2+^. The resulting normalized climate change (CC) impacts were 8.44, 31.35, and 43.05 kg CO_2_-eq kg^−1^ metal uptake, while the corresponding cumulative energy demands (CED) were 205, 761, and 1045 MJ kg^−1^ metal uptake for Pb^2+^, Cu^2+^, and Zn^2+^, respectively (Figure 2).

The differences among the three metals were governed by their adsorption capacities. ANMF exhibited the highest adsorption capacity for Pb^2+^ (683.8 mg g^−1^) [32], resulting in the lowest normalized CC and CED. In contrast, the lower adsorption capacities for Cu^2+^ (184.1 mg g^−1^) and Zn^2+^ (134.0 mg g^−1^) increased the amount of ANMF required to remove 1 kg of metal, leading to proportionally higher normalized production burdens. Accordingly, the CC impact for Cu^2+^ removal was approximately 3.7 times higher than that for Pb^2+^ removal, while Zn^2+^ removal was approximately 5.1 times higher. A similar trend was observed for CED because the same production inventory was normalized using different adsorption capacities.

These results show that the environmental burden per unit of pollutant uptake is controlled not only by the production impact of the adsorbent but also by its pollutant-specific removal performance [17,18]. Therefore, a single production-based value for ANMF cannot fully represent its environmental performance across different target contaminants. The same adsorbent can appear substantially more or less environmentally favorable depending on the pollutant considered, emphasizing the need to evaluate adsorbents under the same theoretical metal-uptake basis rather than solely on a mass-production basis.

**Figure 3:**
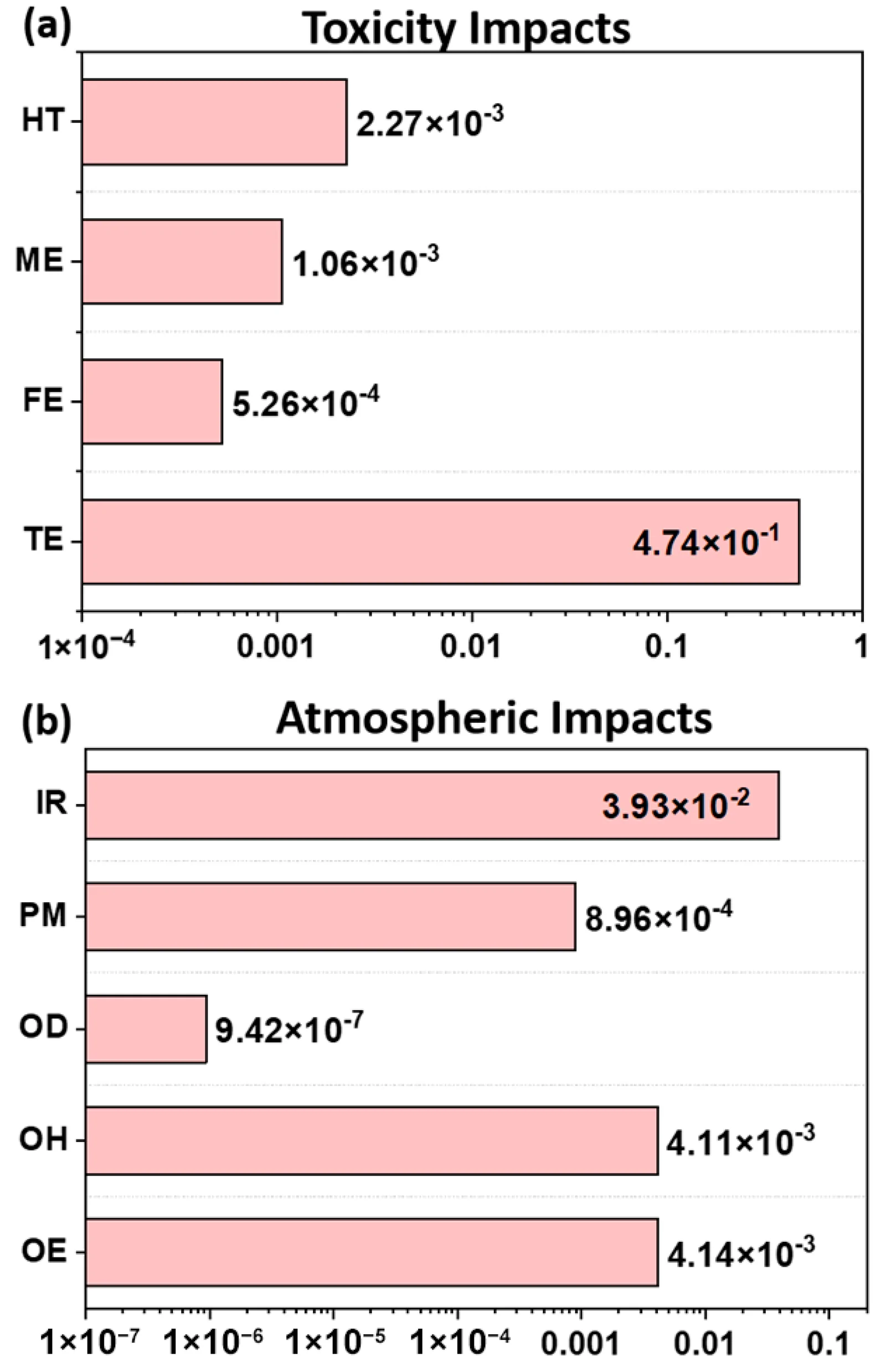
Normalized toxicity and atmospheric environmental impacts associated with ANMF production for the removal of 1 kg of Pb^2+^: (**a**) toxicity-related impact categories and (**b**) atmospheric impact categories.

**Figure 4:**
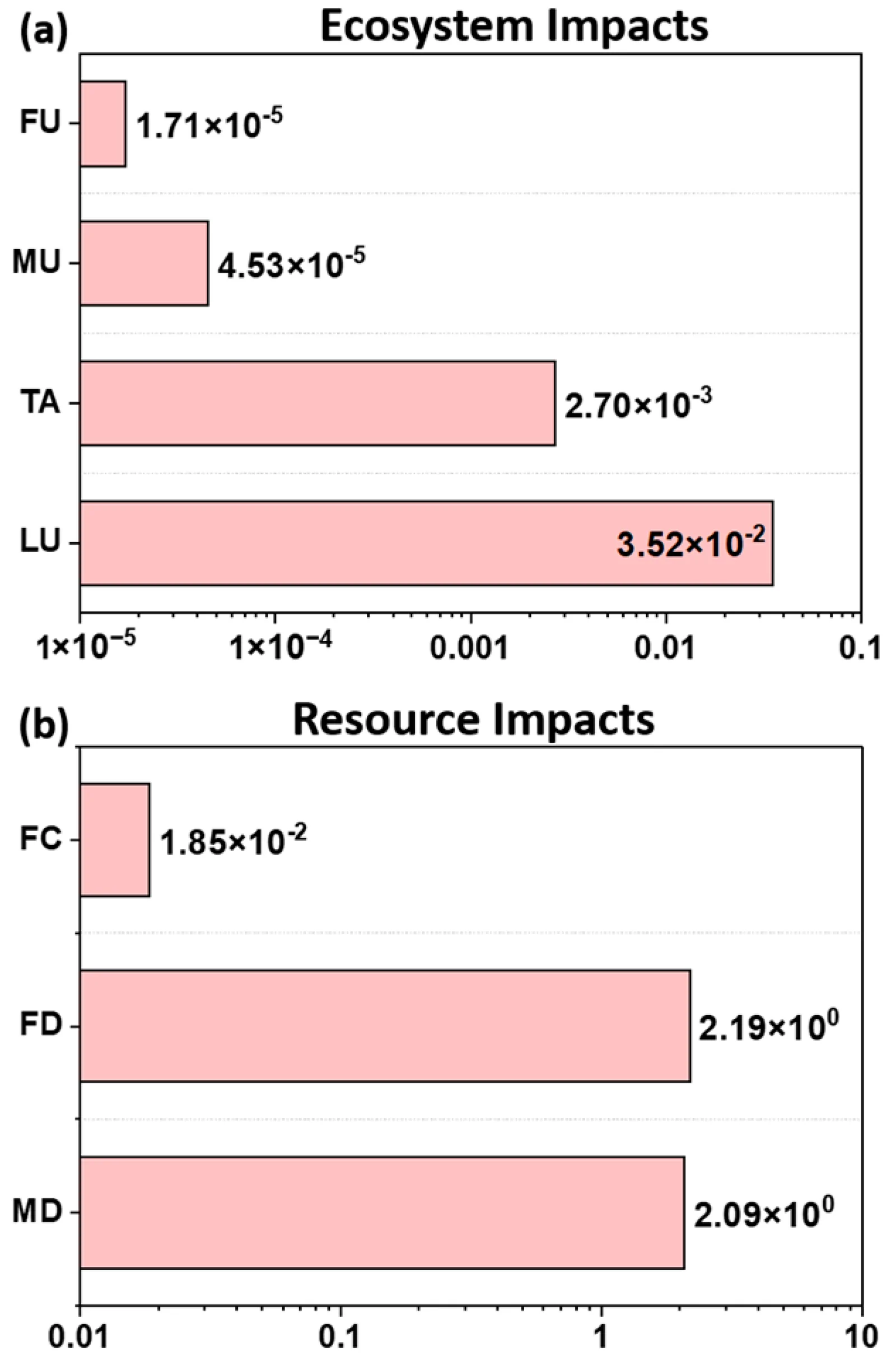
Normalized ecosystem and resource-related environmental impacts associated with ANMF production for the removal of 1 kg of Pb^2+^: (**a**) ecosystem-related impact categories and (**b**) resource-related impact categories.

**Figure 5:**
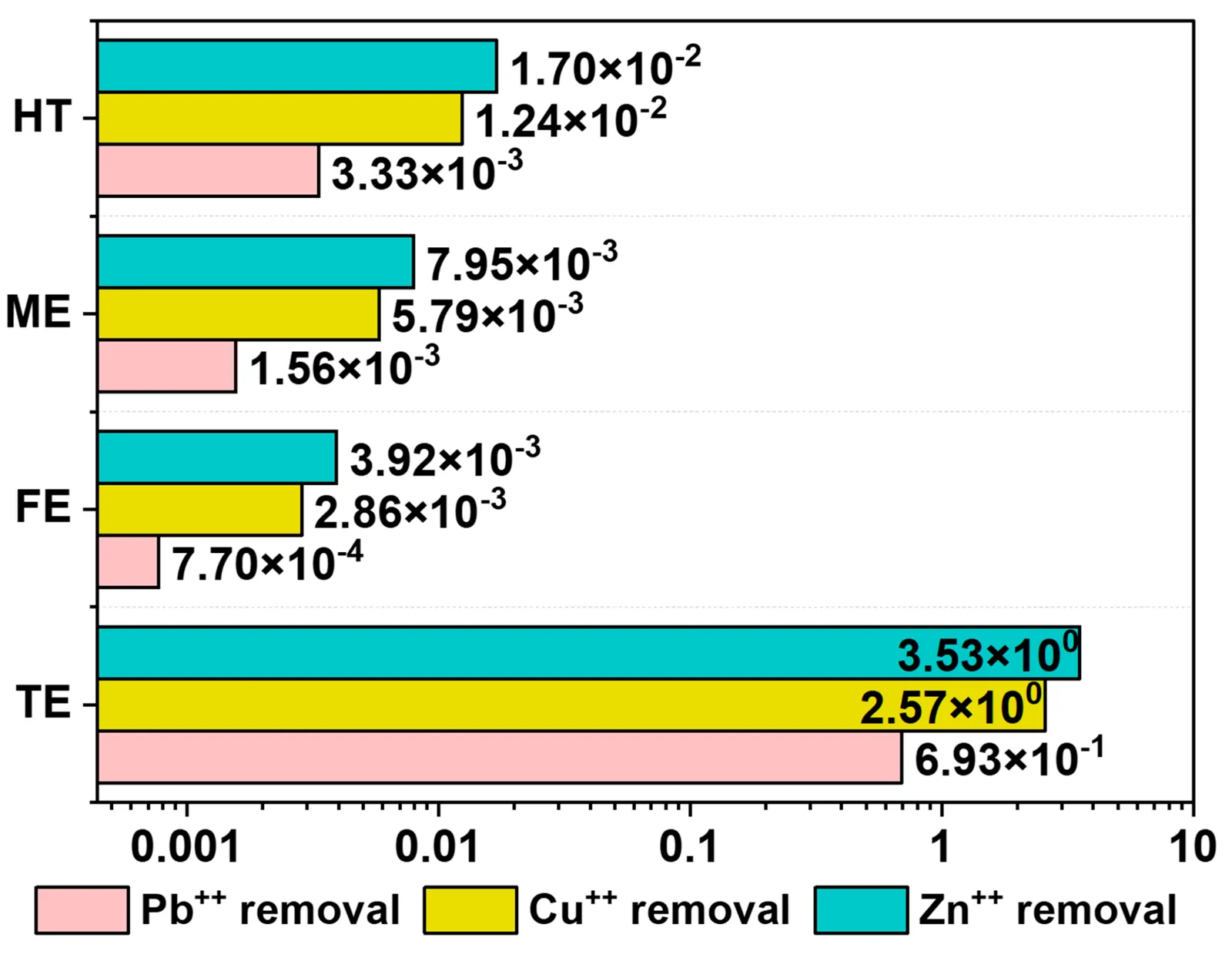
Normalized toxicity-related environmental impacts associated with the removal of 1 kg of Pb^2+^, Cu^2+^, and Zn^2+^ using ANMF.

The adsorption-capacity-normalized trend observed for CC and CED was also reflected across the other evaluated impact categories, i.e., toxicity-related, atmospheric, ecosystem, and resource-related impact categories (Figure 3, Figure 4 and Figure 5). The category-specific results for Pb^2+^ normalization are shown in Figure 3 and Figure 4. Because ReCiPe midpoint indicators are expressed using category-specific characterization units, their absolute numerical magnitudes should not be compared directly across categories. The figures are therefore used to report the calculated category-specific results, while interpretation focuses on changes within the same impact category across the metal-normalization scenarios. As a reference for interpreting the magnitude of these midpoint results, the human carcinogenic toxicity value of 2.27 × 10^−3^ kg 1,4-DCB eq for the Pb^2+^-normalized case can be compared with the ReCiPe 2016 World (2010) [33] H normalization reference of approximately 10.298 kg 1,4-DCB eq per capita. The calculated value corresponds to approximately 2.20 × 10^−4^ of the annual world-average per capita reference, or about 0.022%. This comparison provides a relative scale for interpretation but should not be regarded as a regulatory safety threshold or a direct estimate of human-health risk [33].

These differences reflect the upstream material and energy requirements associated with ANMF production, particularly chemical activation, electricity consumption, and process energy use.

When the same impact categories were normalized for Cu^2+^ and Zn^2+^ removal, the ranking among metals remained consistent with the adsorption-capacity trend (Figure 5). Pb^2+^ removal showed the lowest toxicity-related burdens because ANMF exhibited the highest Pb^2+^ adsorption capacity, whereas Zn^2+^ removal showed the highest burdens because of its lower adsorption capacity. For example, terrestrial ecotoxicity increased from 0.693 for Pb^2+^ removal to 2.57 and 3.53 for Cu^2+^ and Zn^2+^ removal, respectively. Similarly, human toxicity increased from 0.00333 for Pb^2+^ to 0.0124 for Cu^2+^ and 0.017 for Zn^2+^. This proportional increase confirms that the normalized production burden is strongly governed by the amount of adsorbent required to remove a fixed quantity of metal.

These results indicate that the influence of adsorption performance is not limited to climate change and process energy demand. Instead, adsorption capacity affects all impact categories when impacts are expressed per unit of pollutant uptake. Therefore, relying only on production-based indicators may obscure important differences in normalized environmental performance, particularly when adsorbents exhibit metal-specific adsorption capacities. This finding is consistent with recent performance-oriented LCA approaches for adsorbents, which emphasize that adsorption performance and environmental impacts should be considered together when comparing adsorbent sustainability [17,18].

### 2.3. Integrated Comparison of Production and Adsorption-Capacity-Normalized Impacts

**Figure 6:**
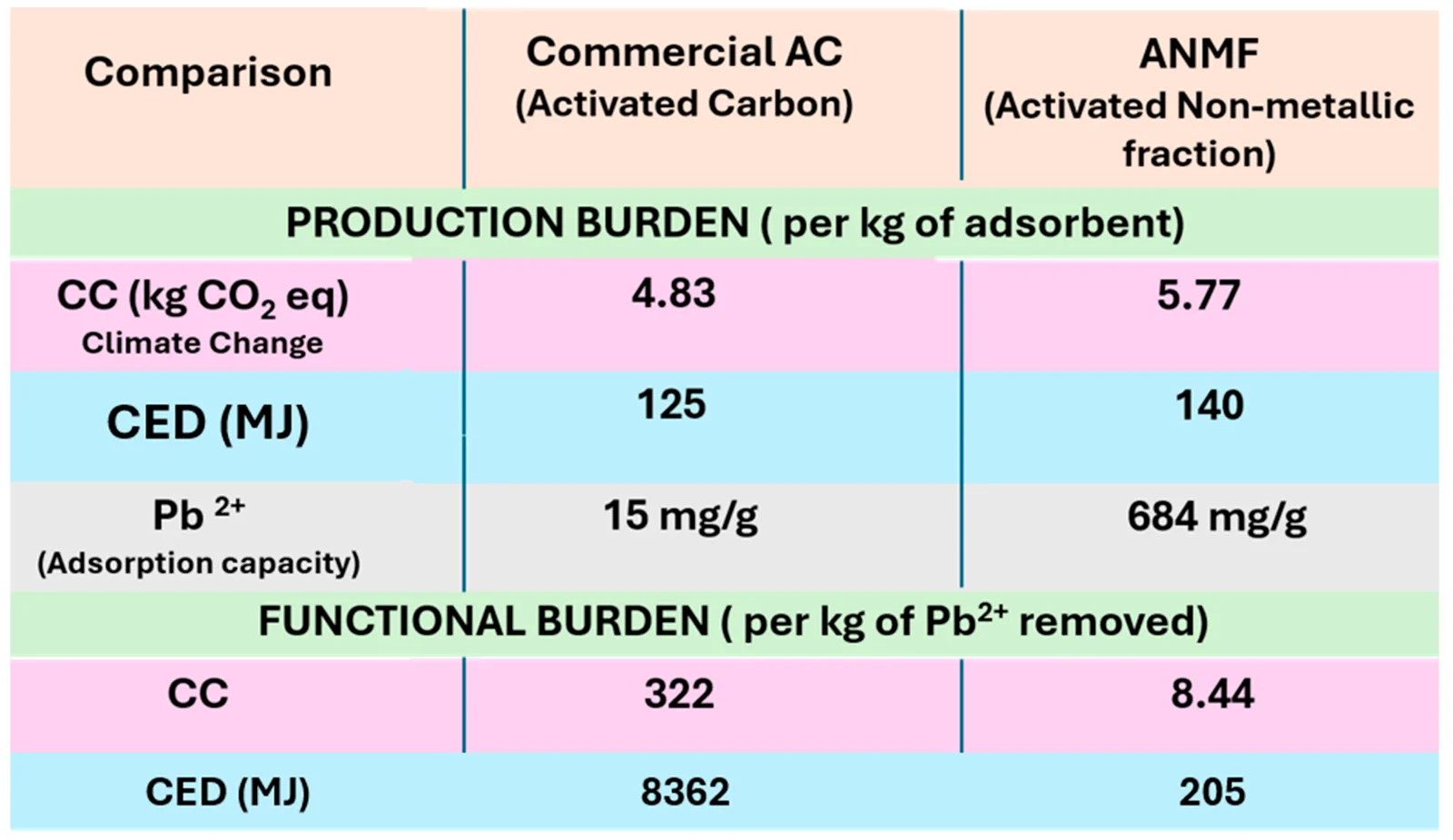
Integrated comparison of production-based impacts per kilogram of adsorbent and adsorption-capacity-normalized production impacts per kilogram of theoretical Pb^2+^ uptake for commercial AC and ANMF.

To examine how adsorption performance influences the production-based comparison, ANMF was benchmarked against commercial AC, which is widely used for heavy-metal removal. A representative Pb^2+^ adsorption capacity of 15 mg g^−1^ for commercial AC was established from literature data (Table 1). The production-based and normalized environmental performances of both adsorbents are summarized in Table 2 and illustrated in Figure 6.

On a production basis, commercial AC exhibited slightly lower environmental burdens than ANMF, with climate change (CC) impacts of 4.83 and 5.77 kg CO_2_-eq kg^−1^ adsorbent and cumulative energy demands (CED) of 125.43 and 140.03 MJ kg^−1^ adsorbent, respectively. This indicates that, when evaluated only per kilogram of adsorbent produced, commercial AC appears marginally more favorable than ANMF. However, this production-based comparison does not account for the large difference in Pb^2+^ adsorption capacity between the two materials. Commercial AC had a representative Pb^2+^ adsorption capacity of 15 mg g^−1^, whereas ANMF exhibited a substantially higher capacity of 683.8 mg g^−1^.

When the production impacts were normalized to the same theoretical Pb^2+^ uptake basis, the environmental comparison changed markedly. Because commercial AC requires a much larger quantity of adsorbent to remove 1 kg of Pb^2+^, its normalized CC and CED increased to 322.0 kg CO_2_-eq and 8362 MJ kg^−1^ Pb^2+^ uptake, respectively. In contrast, the corresponding normalized burdens for ANMF were only 8.44 kg CO_2_-eq and 205 MJ kg^−1^ Pb^2+^ uptake. Thus, although ANMF has slightly higher production-related impacts per kilogram of adsorbent, its higher Pb^2+^ uptake capacity substantially reduces the environmental burden associated with achieving the same removal target.

This comparison demonstrates the importance of considering adsorption performance alongside production impacts when evaluating adsorbents. Small differences in manufacturing-related impacts may become less important when adsorption capacities differ by one or more orders of magnitude. Therefore, production-based comparisons alone may not fully represent the environmental performance of adsorbents intended for wastewater treatment. The integrated comparison in Figure 6 shows that performance-based normalization complements conventional mass-based LCA by enabling adsorbents to be compared under equivalent heavy-metal removal conditions.

Direct comparison of adsorption capacities reported across different studies is affected by differences in pH, initial concentration, temperature, adsorbent dosage, contact time, and water matrix. Consequently, the compiled commercial-AC capacities are used as a literature benchmarking distribution rather than as evidence of a single intrinsic Pb^2+^ capacity for commercial activated carbon.

### 2.4. Sensitivity Analysis

Two complementary sensitivity analyses were conducted to examine uncertainty in the cradle-to-gate production inventory and in the subsequent adsorption-capacity normalization. First, selected foreground inventory parameters were varied independently while all other inputs were kept constant. As summarized in Table 3, electricity and KOH consumption exerted the greatest influence on the production impacts. A ±20% variation in total process electricity changed CC by approximately ±10.6% and CED by ±9.5%, whereas a ±20% variation in KOH consumption changed CC by approximately ±9.3% and CED by ±10.3%. These results are consistent with the contribution analysis in Section 3.1, which identified electricity and KOH as the dominant contributors to both indicators.

In comparison, transport and washing-related parameters had substantially smaller effects. Increasing the transport distance from the base value of approximately 30 km to 300 km increased CC by only 1.2% and CED by 0.7%. Similarly, varying washing-water consumption and the associated wastewater-treatment load by ±25% changed both indicators by less than 0.2%. Therefore, within the ranges evaluated, uncertainty in electricity and KOH consumption is considerably more influential than uncertainty associated with transport, washing-water supply, or wastewater treatment.

A second sensitivity analysis examined the effect of utilizing only a fraction of the reported equilibrium adsorption capacity (Table 4). Reducing capacity utilization from 100% to 75% increased the performance-normalized production impacts by approximately 33.3%, whereas 50% utilization doubled the corresponding CC and CED values. The corresponding CED values at 50% capacity utilization were 410, 1521, and 2090 MJ kg^−1^ metal uptake for Pb^2+^, Cu^2+^, and Zn^2+^, respectively. Although the relative order of Pb^2+^, Cu^2+^, and Zn^2+^ remained unchanged, the absolute normalized production burdens increased substantially as capacity utilization decreased. These scenarios should be interpreted as bounding cases for uncertainty in practical adsorption performance rather than as experimentally measured column or breakthrough capacities. The results therefore emphasize the importance of obtaining representative working-capacity data when extending the present cradle-to-gate assessment toward a treatment-level LCA.

### 2.5. Scope Limitations and Implications for Wastewater-Treatment Applications

The present cradle-to-gate assessment ends at the production of dry ANMF and therefore excludes regeneration and management of metal-loaded adsorbent. Following adsorption, ANMF containing Pb^2+^, Cu^2+^, or Zn^2+^ would require regeneration and reuse or controlled management as a metal-containing waste. These post-gate processes may involve additional chemicals, water and energy consumption, recovered-metal credits, and disposal-related impacts. Consequently, the performance-normalized production impacts reported here should not be interpreted as cradle-to-grave wastewater-treatment impacts.

Normalization to 1 kg of metal uptake should not be interpreted as implying that removal of 1 kg of Pb^2+^, Cu^2+^, and Zn^2+^ represents an equivalent wastewater-treatment service. Influent concentrations, regulatory discharge limits, competing ions, and water-matrix characteristics differ among contaminants. A treatment-oriented assessment could instead use a functional unit such as 1 m^3^ of wastewater treated from a defined initial concentration to a specified discharge concentration; however, such an assessment would require inclusion of use-stage energy, chemical dosing, separation, regeneration, and end-of-life management.

The cut-off treatment of recovered NMF also excludes upstream WPCB processing and corona-electrostatic separation burdens. Alternative allocation approaches could be assessed in future work if a defensible allocation basis for the recovered NMF stream becomes available.

## 3. Materials and Methods

### 3.1. Goal, Scope, and Assessment Framework

The goal of this study was to quantify the cradle-to-gate environmental impacts associated with the production of activated non-metallic fraction (ANMF) from recovered waste printed circuit board non-metallic fraction (WPCB-NMF) and to examine how adsorption capacity influences the interpretation of these production burdens. The primary production reference was 1 kg of dry ANMF produced at the production gate. The cradle-to-gate system boundary terminates at dry ANMF production and does not include wastewater-treatment use-stage operations, such as pH adjustment, mixing or pumping, solid–liquid separation, regeneration, or management of spent adsorbent. As a secondary performance indicator, the cradle-to-gate production impacts were normalized using metal-specific equilibrium adsorption capacities. These normalized indicators therefore do not represent a complete LCA of the wastewater-treatment service. The production route was based on the potassium hydroxide (KOH)-assisted thermochemical conversion process previously developed for transforming WPCB-derived NMF into functional ion-exchange adsorbents for heavy-metal removal [46]. In the original process, NMF recovered after corona electrostatic separation was impregnated with KOH at an NMF:KOH mass ratio of 1:2, thermochemically converted at 250 °C for 3 h under a nitrogen atmosphere, washed to neutral pH, and subsequently dried to obtain the activated non-metallic fraction (ANMF) adsorbent.

WPCB-derived NMF was treated as a recovered waste stream entering the foreground system with zero upstream environmental burden according to the cut-off approach. No burden from PCB production or preceding metal-recovery operations was allocated to NMF, and no credit for avoided landfill or incineration was assigned. Transport of the recovered NMF to ANMF production was included.

Previous investigations demonstrated that the NMF:KOH mass ratio strongly influences pore development, mesoporosity, and ion-exchange-site formation, with an NMF:KOH ratio of 1:2 identified as an effective condition for producing highly functional adsorbents [47]. Potassium hydroxide (KOH, >85%, Sigma-Aldrich, St. Louis, MO, USA) was used in the previously reported activation process. Consequently, the NMF:KOH ratio of 1:2 was retained throughout the present study.

**Figure 7:**
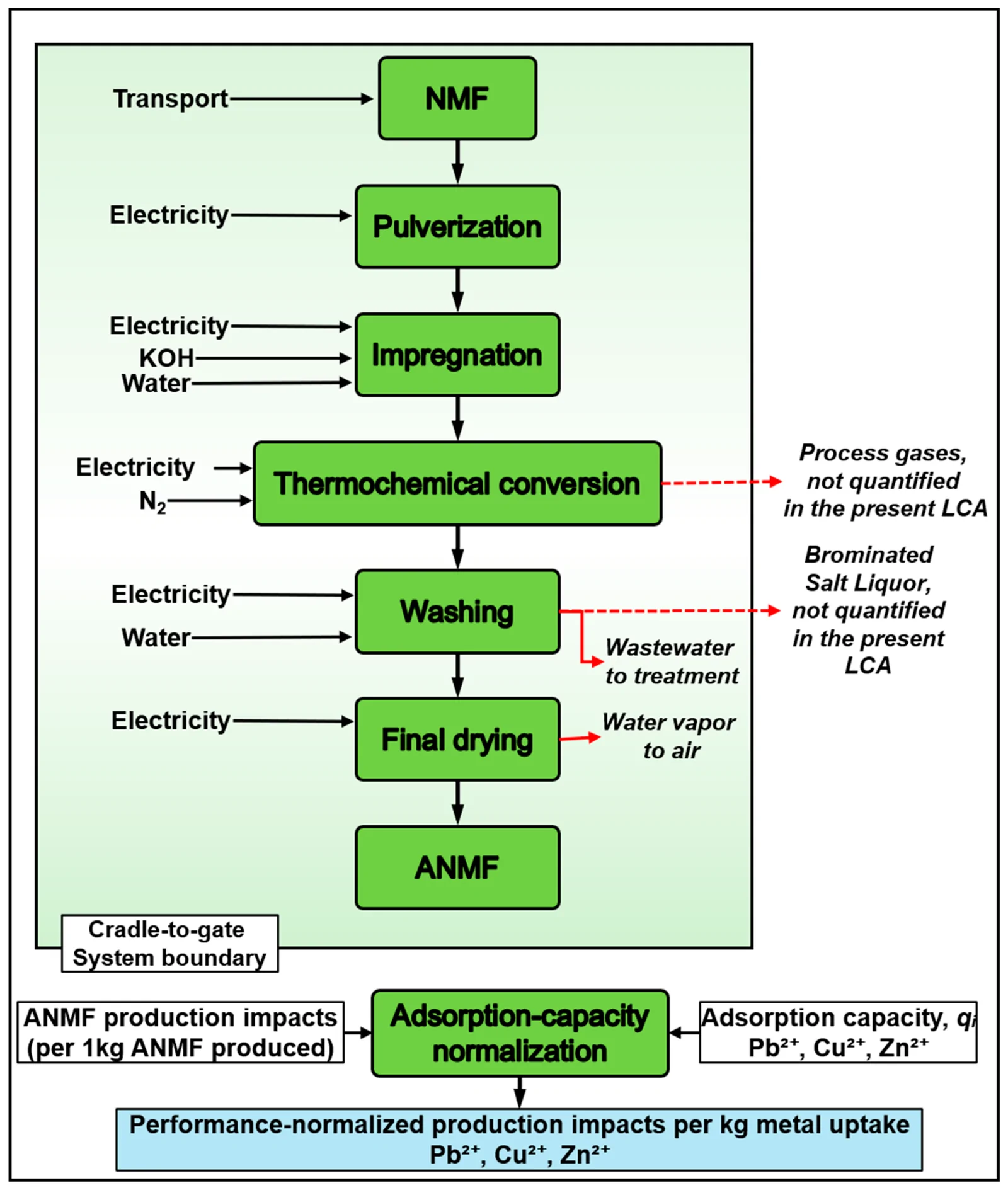
Cradle-to-gate system boundary for the production of adsorbent.

For foreground life-cycle inventory development, a prospective concentrated-paste configuration was modelled by reducing the mixing-water requirement while retaining the NMF:KOH ratio of 1:2. The modelled NMF:KOH:water mass ratio was 1:2:3, compared with the more dilute 1:2:35 preparation condition reported previously. The 1:2:3 configuration is therefore an LCA modelling assumption and has not been experimentally demonstrated to produce identical adsorption performance. The uncertainty associated with applying the published equilibrium adsorption capacities to this prospective production configuration is acknowledged and is partially explored through the adsorption-capacity-utilization scenarios. The overall assessment framework is illustrated in Figure 7, and the corresponding foreground life-cycle inventory is presented in Table 5.

The assessment consisted of four sequential stages. First, the cradle-to-gate inventory for ANMF production was established. Second, previously reported equilibrium adsorption capacities for Pb^2+^, Cu^2+^, and Zn^2+^ were compiled. Third, the production-related environmental impacts were normalized using the corresponding adsorption capacities and expressed per kilogram of theoretical metal uptake. Finally, the adsorption-capacity-normalized production impacts of ANMF were benchmarked against commercial AC using Pb^2+^ adsorption-performance data reported in the literature.

### 3.2. Production Inventory

The foreground life-cycle inventory (LCI) was developed for the concentrated-paste production route described in Section 2.1. The production system comprised pulverization of the waste printed circuit board (WPCB) non-metallic fraction (NMF), impregnation with potassium hydroxide (KOH), thermochemical conversion, washing, and final drying to produce the activated non-metallic fraction (ANMF) adsorbent. The foreground inventory included all material and energy inputs associated with adsorbent production, including waste NMF, KOH, process water, electricity, nitrogen, transportation, and wastewater treatment. The complete foreground inventory used in this study is presented in Table 5.

Background processes were obtained from the GaBi database using consistent datasets, system boundaries, and modelling assumptions. Washing wastewater was represented using the Sphera dataset “DE: Municipal wastewater treatment (mix); mechanical and biological treatment; production mix, at plant; 50% agricultural sludge application, 50% sludge incineration.” KOH participates in the activation process, while residual potassium-containing soluble species, alkalinity, and soluble reaction products are transferred to the washing effluent. Because detailed experimental speciation of the wastewater was unavailable, the selected wastewater-treatment process was used as a generic treatment proxy. Process gases generated during thermochemical conversion and the brominated salt liquor generated during washing were identified as process outputs but were not quantitatively included in the present LCA because their compositions and treatment requirements were not sufficiently characterized. These streams are therefore shown as unquantified outputs in Figure 7.

### 3.3. Adsorption Performance

The adsorption capacities used for normalization were obtained from previously published equilibrium adsorption experiments on WPCB-NMF-derived activated ion-exchange material. The equilibrium tests used 0.05 g of adsorbent in 50 mL single-metal solutions, corresponding to an adsorbent dosage of 1 g L^−1^, with initial metal concentrations ranging from 10 to 1000 mg L^−1^ and agitation at 120 rpm. Equilibrium was observed after approximately 5 d, while the equilibrium-isotherm experiments were maintained for 10 d. The reported maximum equilibrium capacities used in the present study were 683.8, 184.1, and 134.0 mg g^−1^ for Pb^2+^, Cu^2+^, and Zn^2+^, respectively. These values were used only as equilibrium performance indicators for adsorption-capacity normalization and should not be interpreted as practical column or breakthrough capacities [34].

The adsorption capacities were converted to kilograms of heavy metal uptake per kilogram of adsorbent and subsequently used to normalize the production-related environmental impacts. The adsorption capacities were converted to kilograms of metal uptake per kilogram of adsorbent and used to normalize the corresponding cradle-to-gate production impacts. The resulting indicators are therefore expressed per kilogram of theoretical metal uptake and describe the influence of adsorption capacity on the interpretation of the same ANMF production inventory.

### 3.4. Adsorption-Capacity Normalization of Production Impacts

Conventional LCAs of adsorbents typically express environmental impacts per kilogram of adsorbent produced. While this approach quantifies the environmental burden associated with material production, it does not account for adsorption performance, which determines the amount of adsorbent required to achieve a specific treatment objective.

The primary reference for the cradle-to-gate assessment was 1 kg of dry ANMF produced. As a secondary performance indicator, the resulting production impacts were normalized by the metal-specific adsorption capacity. The normalized production impact for metal i was calculated according to Equation (1):

(1) $$PNI_{i}=\frac{PI}{q_{i}}$$

where *PNI_i_* = performance-normalized production impact for metal *i*, *PI* = cradle-to-gate production impact per kg ANMF, and *q_i_* = the adsorption capacity expressed as kilograms of metal uptake per kg ANMF.

The resulting values represent cradle-to-gate production burdens normalized by adsorption capacity and should not be interpreted as the environmental impact of a complete wastewater-treatment service.

The same normalization procedure was applied to all evaluated environmental impact categories and to CED. The resulting indicators describe how metal-specific equilibrium adsorption capacity changes the interpretation of cradle-to-gate ANMF production burdens.

### 3.5. Benchmarking Against Commercial Activated Carbon

Commercial AC was selected as the benchmark material because it is the most widely applied adsorbent for heavy-metal removal in water treatment. Reported Pb^2+^ adsorption capacities of commercial AC were compiled from the literature (Table 1). The values ranged from 5.20 to 162 mg g^−1^, with a median of 15.86 mg g^−1^ and an arithmetic mean of 29.50 mg g^−1^. A representative value of 15 mg g^−1^, approximately corresponding to the median, was retained for the base comparison. The mean capacity was additionally evaluated as an alternative performance scenario.

Production-related climate change (CC) and cumulative energy demand (CED) for commercial AC were obtained from the GaBi database using the same life-cycle impact assessment methodology, background datasets, and modelling assumptions applied to ANMF. Normalized environmental impacts for commercial AC were then calculated using Equation (1), allowing direct comparison of the environmental burdens required to remove the same quantity of Pb^2+^. The commercial AC process represents the closest applicable conventional adsorbent-production dataset available in the Sphera/GaBi database; no corresponding database process was available for WPCB-NMF-derived ANMF. The comparison therefore evaluates the production impacts of the two adsorbents on the same theoretical Pb^2+^ uptake basis rather than on adsorbent mass alone.

### 3.6. Life-Cycle Impact Assessment

Environmental midpoint impacts were quantified using the ReCiPe 2016 Midpoint (H) methodology implemented in GaBi/Sphera. Climate change (CC) was selected as the principal emission-related indicator, while cumulative energy demand (CED) was evaluated separately as an energy-use indicator. Additional ReCiPe midpoint categories were assessed to examine toxicity-related, atmospheric, ecosystem, and resource-related impacts. The evaluated impact categories, abbreviations, and characterization units are summarized in Table 6.

ReCiPe midpoint characterization results represent potential environmental impacts and are not regulatory or health-based threshold values. Accordingly, their absolute magnitudes should not be interpreted directly as indicating safe or unsafe levels. For additional context, ReCiPe 2016 provides World (2010) H normalization references based on the average annual environmental burden per person. For human carcinogenic toxicity, the corresponding normalization reference is approximately 10.298 kg 1,4-DCB eq per capita. Normalized values may therefore be used to provide relative scale, while not implying a regulatory threshold or direct human-health risk [33].

The LCA model was developed using GaBi/Sphera [Life Cycle Assessment for Experts (LCAFE) by Sphera/Professional 2024], and the principal background datasets and geographical representations are summarized in Table 7.

Background life-cycle inventories were obtained from the GaBi database using the same datasets, modelling assumptions, and system boundaries adopted for the ANMF production inventory. All impact categories were calculated on both a production basis (per kilogram of adsorbent produced) and a performance basis (per kilogram of heavy metal uptake) using the normalization procedure described in Section 2.4.

### 3.7. Sensitivity Analysis

A one-factor-at-a-time sensitivity analysis was conducted to examine the robustness of the cradle-to-gate production inventory. Total process electricity and KOH consumption were independently varied by ±20%. Transport distance for NMF and KOH was evaluated at the base distance of approximately 30 km and at extended distances of 100 and 300 km. Washing-water supply and the corresponding wastewater-treatment load were varied simultaneously by ±25%. In addition, adsorption-capacity utilization scenarios corresponding to 50%, 75%, and 100% of the reported equilibrium capacities were evaluated to examine the influence of lower practical adsorption performance on the performance-normalized production impacts. All other inventory parameters were held constant in each scenario.

## 4. Conclusions

This study quantified the cradle-to-gate environmental impacts associated with the production of an activated adsorbent from WPCB-derived non-metallic fraction and subsequently normalized these production impacts using metal-specific equilibrium adsorption capacities. Production of 1 kg of ANMF resulted in a CC impact of 5.77 kg CO_2_-eq and a CED of 140.03 MJ. Adsorption-capacity normalization produced different production burdens for Pb^2+^, Cu^2+^, and Zn^2+^ because of their different reported equilibrium uptake capacities.

The contribution analysis showed that process electricity and KOH production dominate the cradle-to-gate impacts, together accounting for approximately 99% of both CC and CED. Sensitivity analysis similarly identified electricity and KOH consumption as the most influential inventory parameters, whereas transport, washing-water supply, and wastewater treatment had comparatively minor effects within the evaluated ranges. Lower adsorption-capacity utilization substantially increased the performance-normalized production impacts, demonstrating the importance of practical adsorption performance when interpreting these results.

Commercial AC exhibited slightly lower production impacts per kilogram of adsorbent, whereas its lower representative Pb^2+^ adsorption capacity resulted in higher production burdens after adsorption-capacity normalization. The comparison therefore demonstrates that production impacts and adsorption performance should be considered together when evaluating adsorbent production pathways.

The reported normalized values represent cradle-to-gate production impacts and do not include wastewater-treatment use-stage operations, regeneration, or spent-adsorbent management. Future treatment-level assessments should incorporate practical column capacities, defined wastewater compositions and discharge targets, operational energy and chemical requirements, regeneration, and end-of-life management.

## Figures and Tables

**Table 1:** Reported Pb^2+^ adsorption capacities of commercial AC used for functional benchmarking.

Reference	Commercial AC Pb^2+^ Capacity (mg g^−1^)	Notes
[34]	5.20	Commercial AC benchmark
[35]	7.55	Commercial bituminous coal-based AC
[36]	7.72	Commercial AC; packed-bed simulation
[37]	9.44	Commercial AC
[38]	12.81	Commercial AC
[39]	13.06	Commercial AC in column/filter study
[40]	18.65	Converted from 90 μmol g^−1^
[41]	21.69	Commercial AC
[42]	26.30	Commercial AC benchmark
[43]	27.03	Commercial AC
[44]	42.50	Commercial AC benchmark
[45]	162	Commercial microporous AC *

* WG-12 is a commercial AC with a high specific surface area (1100 m^2^ g^−1^) and the highest concentration of acidic surface functional groups among the ACs evaluated.

**Table 2:** Production-based and adsorption-capacity-normalized environmental impacts of ANMF and commercial AC for Pb^2+^ uptake.

Adsorbent	Production CC	CED	Pb Capacity	Normalized CC	Normalized CED
Units	(kg CO_2_-eq/kg Adsorbent)	(MJ/kg Adsorbent)	(mg/g)	(kg CO_2_-eq/kg Pb Uptake)	(MJ/kg Pb Uptake)
Commercial AC	4.83	125.43	15	322.0	8362
Commercial AC, mean-capacity scenario	4.83	125.43	29.50	163.7	4252
ANMF	5.77	140.03	683.8	8.44	204.8

CC = climate change; AC = activated carbon; ANMF = activated non-metallic fraction; CED = cumulative energy demand; Pb = lead.

**Table 3:** One-factor-at-a-time sensitivity analysis of cradle-to-gate ANMF production impacts.

Scenario	CC (kg CO_2_-eq/kg ANMF)	Change	CED (MJ/kg ANMF)	Change
Base case	5.769	—	140.03	—
Electricity −20%	5.160	−10.56%	126.70	−9.52%
Electricity +20%	6.378	+10.56%	153.36	+9.52%
KOH −20%	5.235	−9.26%	125.63	−10.28%
KOH +20%	6.303	+9.26%	154.43	+10.28%
Transport 100 km	5.787	+0.30%	140.28	+0.18%
Transport 300 km	5.836	+1.16%	140.99	+0.68%
Washing water + WWT −25%	5.759	−0.17%	139.76	−0.19%
Washing water + WWT +25%	5.779	+0.17%	140.30	+0.19%

**Table 4:** Effect of equilibrium adsorption-capacity utilization on performance-normalized production impacts.

Metal	Capacity Utilization	CC kg CO_2_-eq/kg Metal Uptake	CED MJ/kg Metal Uptake
Pb^2+^	100%	8.44	205
Pb^2+^	75%	11.25	273
Pb^2+^	50%	16.87	410
Cu^2+^	100%	31.34	761
Cu^2+^	75%	41.78	1014
Cu^2+^	50%	62.67	1521
Zn^2+^	100%	43.05	1045
Zn^2+^	75%	57.40	1393
Zn^2+^	50%	86.11	2090

**Table 5:** Life-cycle inventory (LCI) of ANMF production.

Process Stage	Inventory Item	Unit	Values
Transport/pulverization	Waste NMF input	kg	1.111
	Transport of NMF	tkm	0.033
	Transport of KOH	tkm	0.066
	Pulverization electricity	kWh	0.111
Impregnation	KOH input	kg	2.222
	Water for mixing	kg	3.333
	Initial slurry/paste mass	kg	6.666
	Stirring electricity	kWh	0.378
	Water entering thermochemical conversion	kg	3.333
	Feed to thermochemical conversion chamber	kg	6.666
Thermochemical conversion	Furnace electricity	kWh	1.387
	Water removed	kg	3.333
	Thermal dehydration electricity	kWh	3.666
	N_2_ input	kg	0.015
Washing	Washing water (20 mL g^−1^ ANMF)	kg	20.00
	Wastewater to treatment	kg	19.70
	Retained water in wet cake	kg	0.300
Final drying	Final drying electricity	kWh	0.330
	Water evaporated	kg	0.300
Product	Dry ANMF	kg	1

Initial slurry/paste mass, water entering thermochemical conversion, and feed to the thermochemical conversion chamber are intermediate process flows and are not additional material inputs. Process gases and brominated salt liquor were identified as process outputs but were not quantitatively included because their compositions and treatment requirements were not sufficiently characterized. Total process electricity = 5.872 kWh kg^−1^ ANMF.

**Table 6:** Environmental impact categories, abbreviations, and their units.

Abbrev.	Environmental Impact Category in ReCiPe 2016 v1.1 Midpoint (H)	Units
CC	Climate change, default, excl biogenic carbon	kg CO_2_ eq.
PM	Fine Particulate Matter Formation	kg PM2.5 eq.
FD	Fossil depletion	kg oil eq.
FC	Freshwater Consumption	m^3^
FE	Freshwater ecotoxicity	kg 1,4 DB eq.
FU	Freshwater Eutrophication	kg P eq.
HT	Human toxicity, cancer	kg 1,4-DB eq.
IR	Ionizing Radiation	kBq Co-60 eq. to air
LU	Land use	Annual crop eq.·y
ME	Marine ecotoxicity	kg 1,4-DB eq.
MU	Marine Eutrophication	kg N eq.
MD	Metal depletion	kg Cu eq.
OE	Photochemical Ozone Formation, Ecosystems	kg NOx eq.
OH	Photochemical Ozone Formation, Human Health	kg NOx eq.
OD	Stratospheric Ozone Depletion	kg CFC-11 eq.
TA	Terrestrial Acidification	kg SO_2_ eq.
TE	Terrestrial ecotoxicity	kg 1,4-DB eq.

**Table 7:** Major background datasets and modelling assumptions used in the LCA.

Input	Dataset Name
Transport	US combination truck, gasoline-powered, USLCI/Sphera
Electricity	DE electricity from natural gas, Sphera
Process water	DE desalinated/deionized water, Sphera
KOH	US potassium hydroxide, Sphera
N_2_	US gaseous nitrogen via cryogenic air separation, Sphera
Wastewater	DE municipal wastewater treatment mix, Sphera
Recovered NMF	Cut-off, zero upstream burden
Commercial AC	DE activated carbon, Sphera

## Data Availability

The original contributions presented in the study are included in the article; further inquiries can be directed to the corresponding authors.
